# Leiomyoma of the Thumb Mimicking a Ganglion: A Case Report

**DOI:** 10.7759/cureus.39295

**Published:** 2023-05-21

**Authors:** Abdullah M AlZahrani, Felwa A AlMarshad, Khalid Fayi, Nora N Alsaud, Jumanah N Alkhater, MHD R Awad, Abdulaziz Jarman

**Affiliations:** 1 Plastic and Reconstructive Surgery Section, Department of Surgery, King Faisal Specialist Hospital and Research Centre, Riyadh, SAU; 2 Plastic and Reconstructive Surgery, King Faisal Specialist Hospital and Research Centre, Riyadh, SAU

**Keywords:** cyst, hand surgery, ganglion cyst, benign hand tumor, leiomyoma

## Abstract

Our case report describes the presence of a leiomyoma in the left-hand thumb of a 69-year-old woman, an extremely uncommon location for such a tumor. Leiomyomas are typically benign tumors that arise from smooth muscle, but their occurrence in the hand is unusual. While leiomyomas are more commonly found in the uterus, they may occasionally develop in the extremities, though this is more frequently observed in the lower limbs. These tumors typically present in patients in their third to fourth decades of life, and they are often not diagnosed until surgery because histological pathology is necessary to confirm the diagnosis.

## Introduction

Leiomyoma is a benign, solitary tumor of the soft tissue that can arise from any area where smooth muscle is present [1]. The uterus is the most common location for leiomyoma, while the lower limbs are more frequently affected than the upper extremities [2,3]. Leiomyomas in the hand are extremely rare [4], likely due to the scarcity of smooth muscle in the area. They can originate from the smooth muscles of the upper limb, such as the walls of blood vessels and sweat glands, and may be linked to vascular structures in the area [4,5]. The differential diagnosis for soft tissue masses in hand includes ganglion cysts, giant cell tumors of the tendon sheath, lipomas, granulomas, glomus tumors, neurofibromas, neurilemmomas, and spindle cell sarcomas [1,4]. Treatment options range from conservative management to surgical excision, depending on the diagnosis. A hand surgeon must be aware of the diagnostic possibilities based on examination. The diagnosis of leiomyoma is typically not conclusive until after surgery, as only histological pathology can confirm it [1,3].

Vascular leiomyomas develop from the tunica media layer of veins [6]. When they occur in the digits, they are usually located on the volar surface, commonly near the base of the neurovascular bundle and never beyond the DIP joint [7]. This distinguishes them from glomus tumors.

Surgical excision is the curative treatment for leiomyoma of the hand. This procedure is generally straightforward and effective, but the tumor may occasionally be located in close proximity to a nerve or vessel, particularly when it is situated on the volar aspect of the hand. In such cases, the use of magnifying loupes is recommended [8,9].

## Case presentation

**Figure 1:**
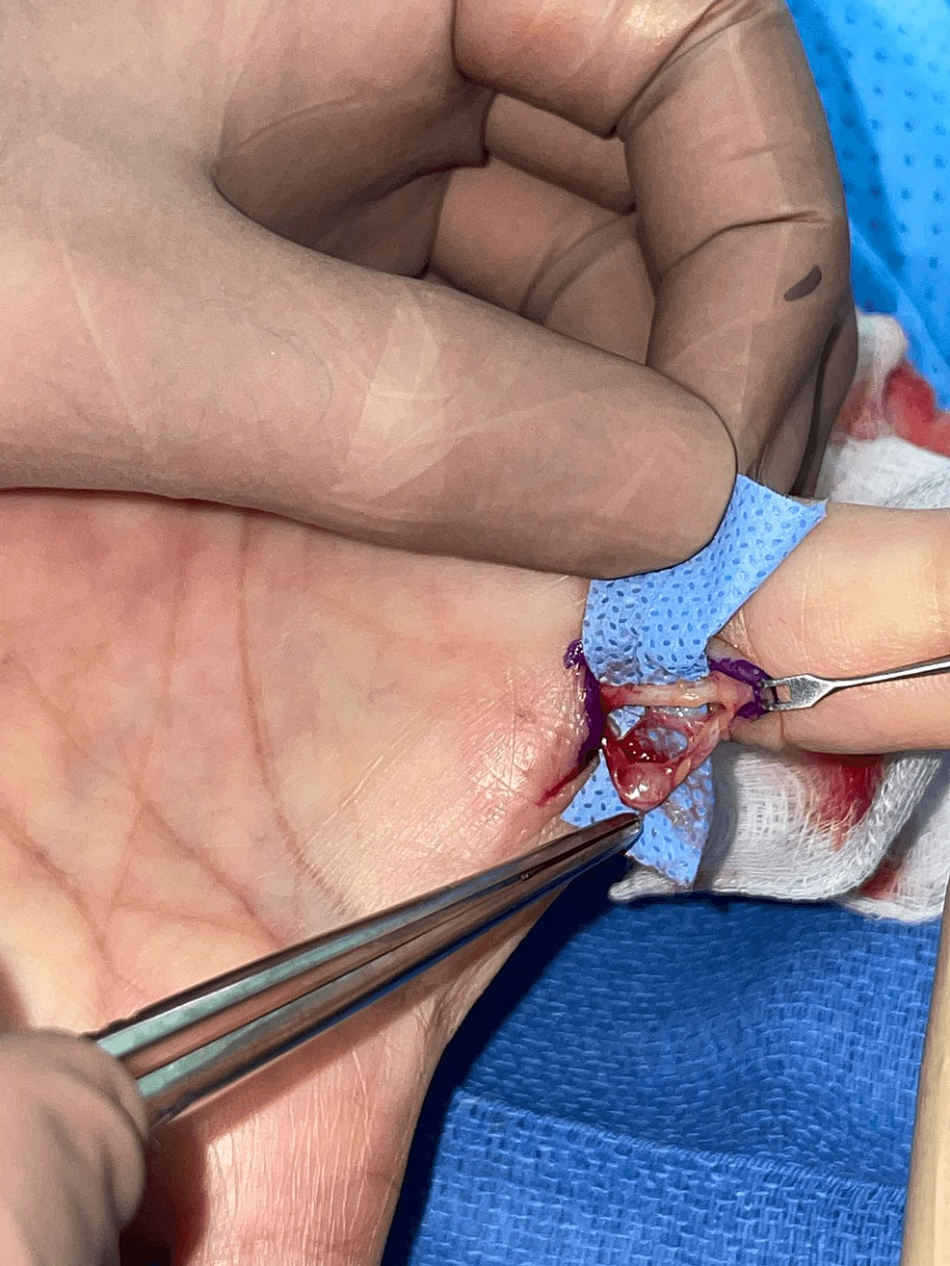
The mass was adhered to the neurovascular pedicle

**Figure 2:**
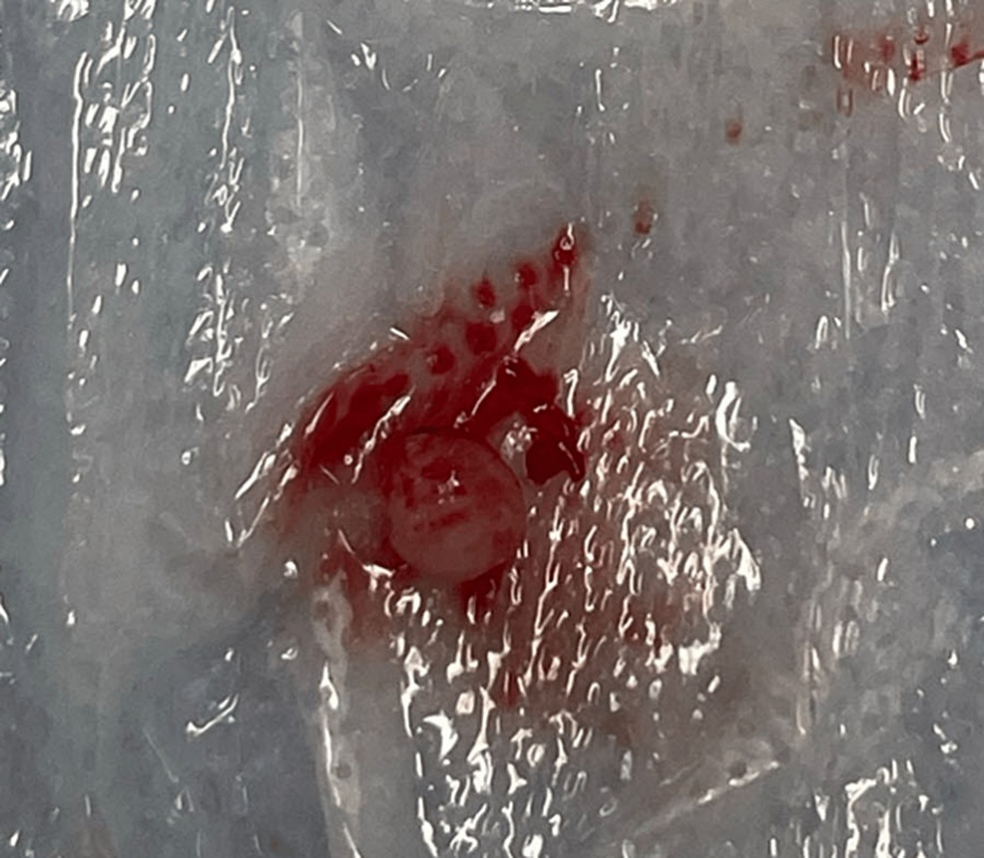
The mass after excision

**Figure 3:**
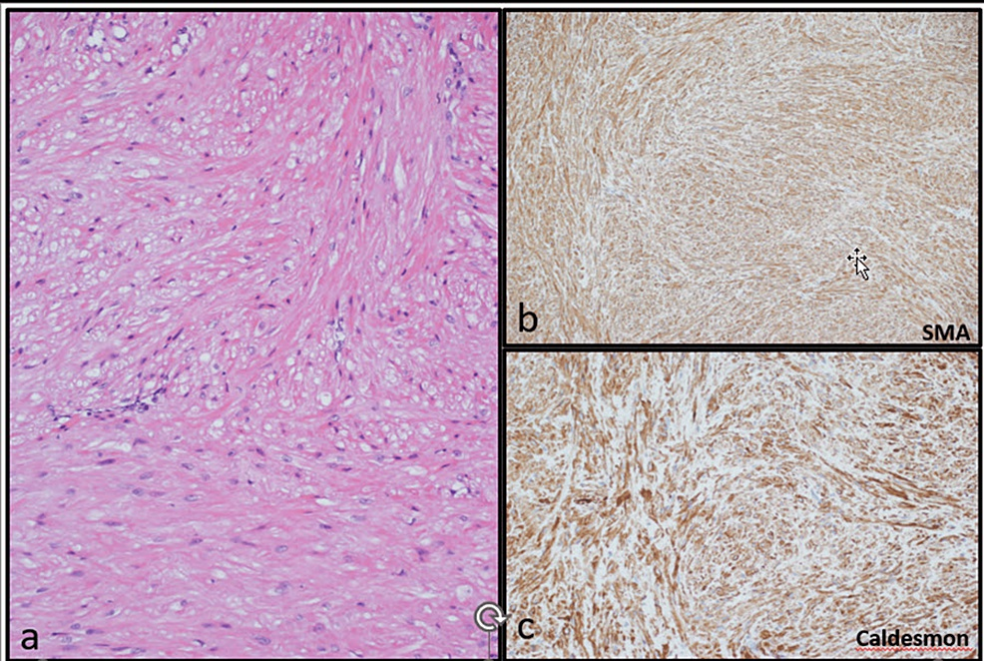
Hematoxylin and eosin-stained section (100x) showing bland spindle cell proliferation with eosinophilic cytoplasm arranged in intersecting fascicles (a) Immunostains for smooth muscle actin (SMA) and caldesmon are diffusely positive in tumor cells (b-c)

A female patient, aged 69 and in good health, visited the clinic due to a painless mass on the volar aspect of her left thumb that had been present for one year. The mass was a single subcutaneous nodule with a firm consistency that gradually increased in size over time but did not affect sensory function. The patient had no history of finger nodules or trauma. Hand X-rays did not show any bone abnormalities in the affected area. Surgical excision was performed with local block anesthesia using an incision over the flexion crease of the thumb. The mass was found to be attached to the flexor sheath and neurovascular pedicle (Figure 1) and measured 0.3 cm × 0.3 cm × 0.3 cm with firm consistency (Figure 2). Microscopic slides revealed bland spindle cell proliferation with eosinophilic cytoplasm arranged in intersecting fascicles. Immunostaining for smooth muscle actin (SMA) and caldesmon was diffusely positive in tumor cells (Figure 3). The final diagnosis was leiomyoma. At the eight-month follow-up, the patient had a healed wound, and both sensory and motor function were intact. No recurrence was observed during the follow-up period.

## Discussion

Leiomyoma, also known as a fibroid, is a benign mesenchymal tumor that commonly grows in areas where smooth muscle is abundant [1]. Such areas include the uterus, urogenital, and gastrointestinal systems. In a surgical review of 560 cases of leiomyoma, over 40% were reported in the lower extremity, and less than 10% were found on the hand [10]. The average age at which patients were reported with leiomyoma of the hand was between 39 and 46 years, with a female to male predominance of a 2:1 ratio [7,11]. In our case report, we present a female patient aged 69 with leiomyoma at an unusual location of the left-hand thumb.

Leiomyomas have been identified into three subtypes that include vascular, cutaneous, and deep soft tissue. Many studies claim that the most common type is vascular leiomyoma, which mainly arises from the tunica media layer of the vein [12]. Although the most typical symptom of leiomyoma is a tenderness that progresses into pain, our patient did not experience any symptoms and only presented with a palpable, non-tender mass at the thumb. The literature suggests theoretically, the mechanism of pain may be due to the tumor being in contact with the peripheral nerve fibers or by active contracture of the tumor's smooth muscle elements, leading to ischemia [12]. Moreover, a substantial association with the leiomyoma of the digit is the trigger finger, especially when connected with the tendon sheath [13]. Around 95% of masses raised on the hand are constituted by ganglion cysts, giant cell tumors of the tendon sheath, epidermoid inclusion cysts, hemangiomas, and lipomas. The remaining minority derives from a variety of uncommon lesions arising from any tissue in the hand [14]. Acknowledging the differential diagnosis is important, as attempts to radically excise the mass may cause extensive neurological deficits. Those to consider are interneural masses that can be easily enucleated (leiomyomas, ganglion cysts, GCTTS, and lipomas) versus those that infiltrate the surrounding tissues (neurofibromas, haemangiomas, and lipofibromatous hamartomas) [14]. Although there has been no specific imaging technique to view leiomyoma, an MRI or US may be useful to better visualize the lesion and its relation to adjacent structures. It can also play a significant role in treatment methods for better surgical planning. A definitive diagnosis of leiomyoma can only be made after surgical excision and histopathologic analysis [9]. The mainstay of treatment is simple surgical excision with ligation of any feeding vessels. If the tumor is properly enucleated and an adequate margin is obtained, the recurrence rate of leiomyoma is low [9,15].

## Conclusions

Leiomyoma is rarely found in the hand, and only a limited number of cases have been reported in the literature. Clinically, it may mimic other more common hand tumors, so it is important to consider other differential diagnoses as they may alter the treatment and surgical approach. Diagnosis is mainly achieved through surgical excision and immunohistochemistry. In our case, a preoperative diagnosis of ganglion cyst was made, and only after excision and postoperative analysis was it confirmed to be leiomyoma.
